# Supplementary material for: Autophagy: A Friend or Foe in Allergic Asthma?
Source: Int J Mol Sci. 2021 Jun 12;22(12):6314. doi: 10.3390/ijms22126314 (PMC8231495; doi:10.3390/ijms22126314)
Supplement: Supplementary file 1 [file ijms-22-06314-s001.zip › ijms-1241289-supplementary.pdf]

International Journal of
Molecular Sciences

MDPI

Review

Autophagy: A Friend or Foe in Allergic Asthma?

Efthymia Theofani [1,2] and Georgina Xanthou [1,*]

1. Cellular Immunology Laboratory, Center for Basic Research; Biomedical Research Foundation of the Academy of Athens, Greece;
2. 1st Department of Respiratory Medicine, "Sotiria" Regional Chest Diseases Hospital, Medical School, National Kapodistrian University of Athens, , Athens, Greece

* Correspondence: gxanthou@bioacademy.gr; tel.: +30 210 65 97 336

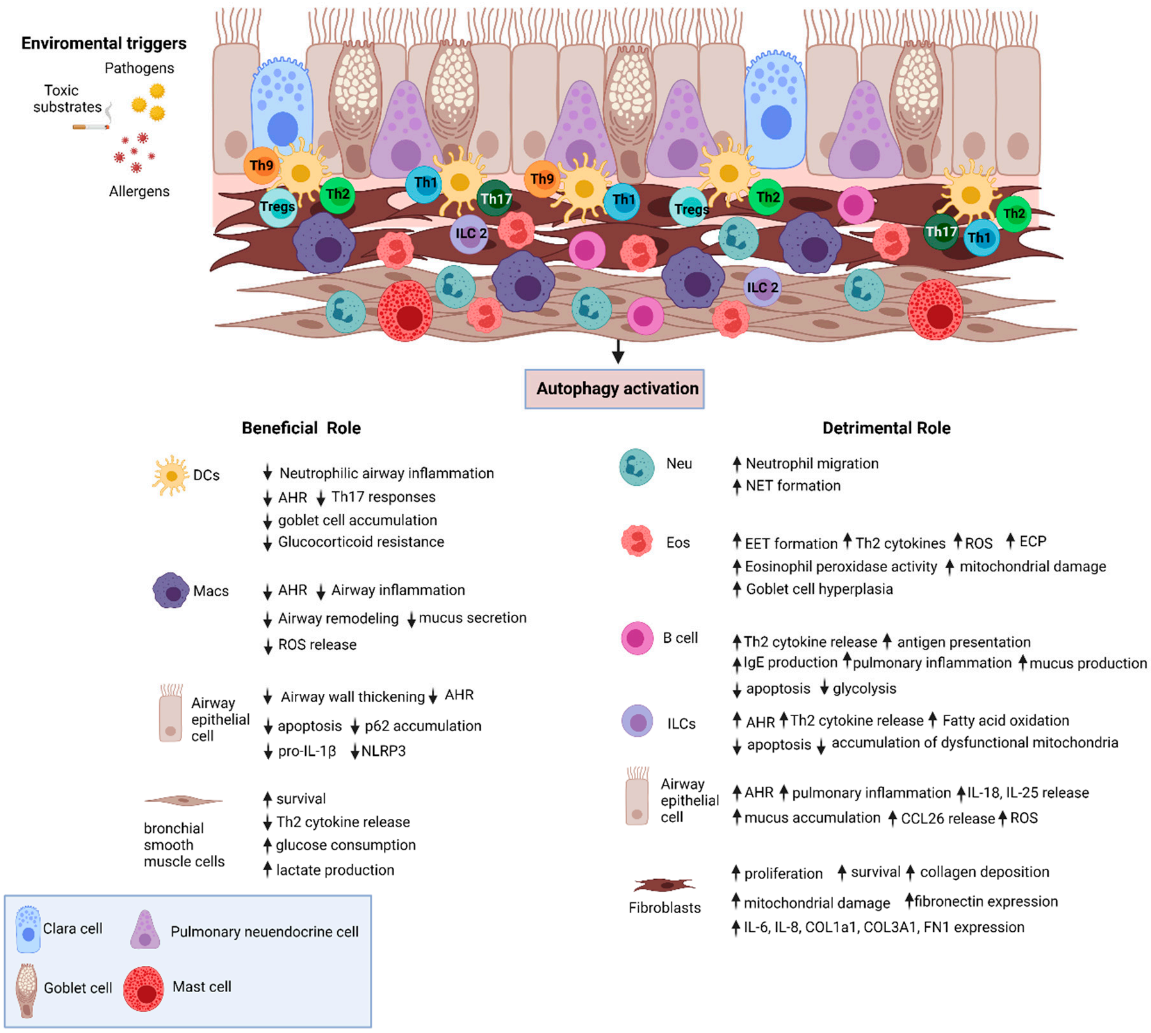

Supplementary Figure S1. Autophagy effects on allergic airway inflammation and remodeling. Exposure to environmental triggers, such as, toxic substrates, pathogens and allergens in the airway activates airway epithelial cells and DCs and induces the infiltration of eosinophils, neutrophils, inflammatory macrophages, B cells and T cells. Antigen-specific Th1, Th2, Th9 and/or Th17 cells produce effector cytokines contributing to AHR, mucus hypersecretion and airway remodeling. The beneficial or detrimental roles of autophagy activation in the context of AAI and airway remodeling are shown.
